# Supplementary material for: Bio-Doped Microbial Nanosilica as Optosensing Biomaterial for Visual Quantitation of Nitrite in Cured Meats
Source: Biosensors (Basel). 2022 Jun 3;12(6):388. doi: 10.3390/bios12060388 (PMC9221271; doi:10.3390/bios12060388)
Supplement: Supplementary file 1 [file biosensors-12-00388-s001.zip › biosensors-1755146-supplementary.pdf]

Supplementary Materials

Bio-Doped Microbial Nanosilica as Optosensing Biomaterial for Visual Quantitation of Nitrite in Cured Meats

Siti Nur Syazni Mohd Zuki [1], Choo Ta Goh [1], Mohammad B. Kassim [2] and Ling Ling Tan [1,*]

[1] Southeast Asia Disaster Prevention Research Initiative (SEADPRI), Institute for Environment and Development (LESTARI), Universiti Kebangsaan Malaysia, UKM Bangi 43600, Selangor Darul Ehsan, Malaysia; p94593@siswa.ukm.edu.my (S.N.S.M.Z.); gohchoota@ukm.edu.my (C.T.G.)

[2] Department of Chemical Sciences, Faculty of Science and Technology, Universiti Kebangsaan Malaysia, UKM Bangi 43600, Selangor Darul Ehsan, Malaysia; mb_kassim@ukm.edu.my

* Correspondence: lingling@ukm.edu.my; Tel.: +60-3-8921-7636; Fax: +60-3-8927-5629

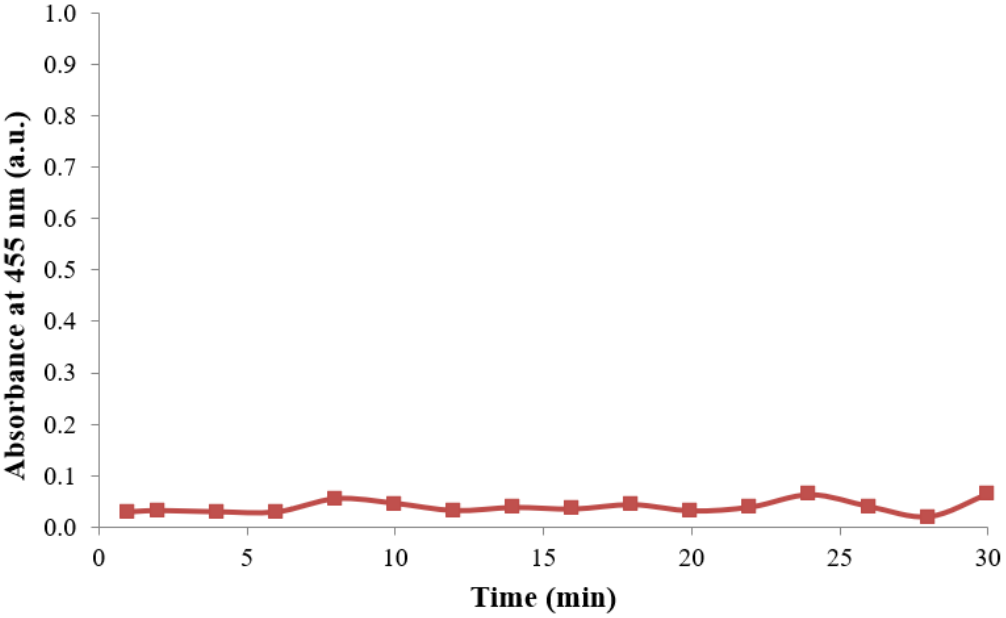

Figure S1. Leaching study of the ruthenium(II) bis(bipyridine)-modified R5-fusion microbial nanosilica in 0.1 M K-phosphate buffer (pH 7.4) over 30 min of experimental period of time.

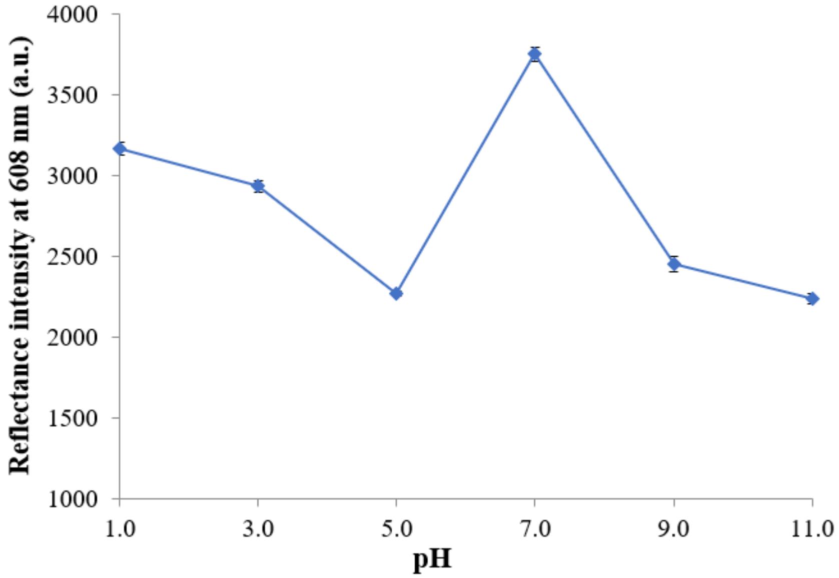

Figure S2. The reflectance response of the microbial optosensor at 608 nm for the determination of 100 mg L^{-1} nitrite in 0.1 M K-phosphate buffer with varying pH conditions from pH 1.0 to pH 11.0 adjusted using 0.1 M NaOH or HCl.

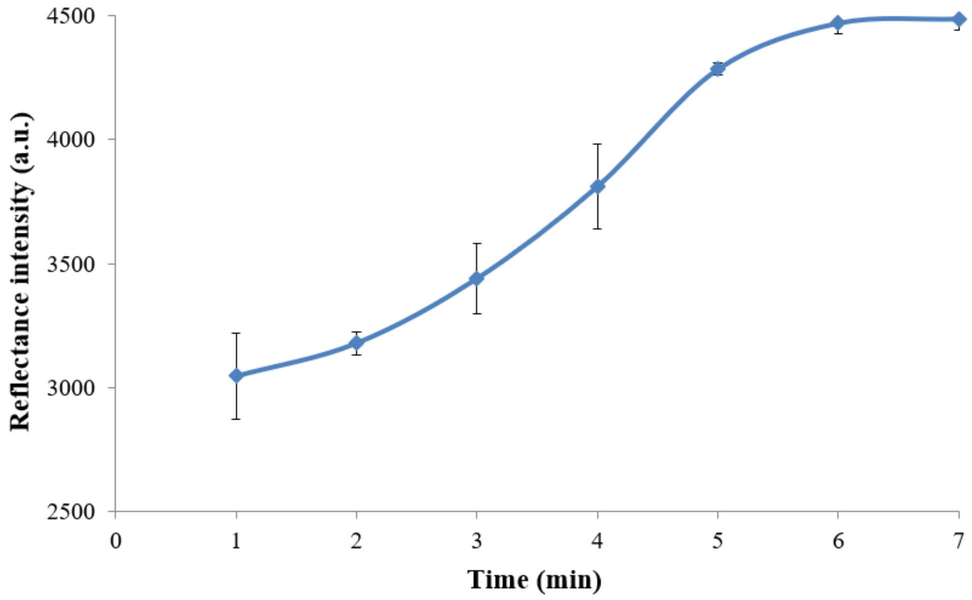

Figure S3. The reflectance microbial biosensor response at 608 nm as a function of enzymatic reaction time for optical detection of 400 mg L^{-1} nitrite in 0.1 M K-phosphate buffer at neutral pH.

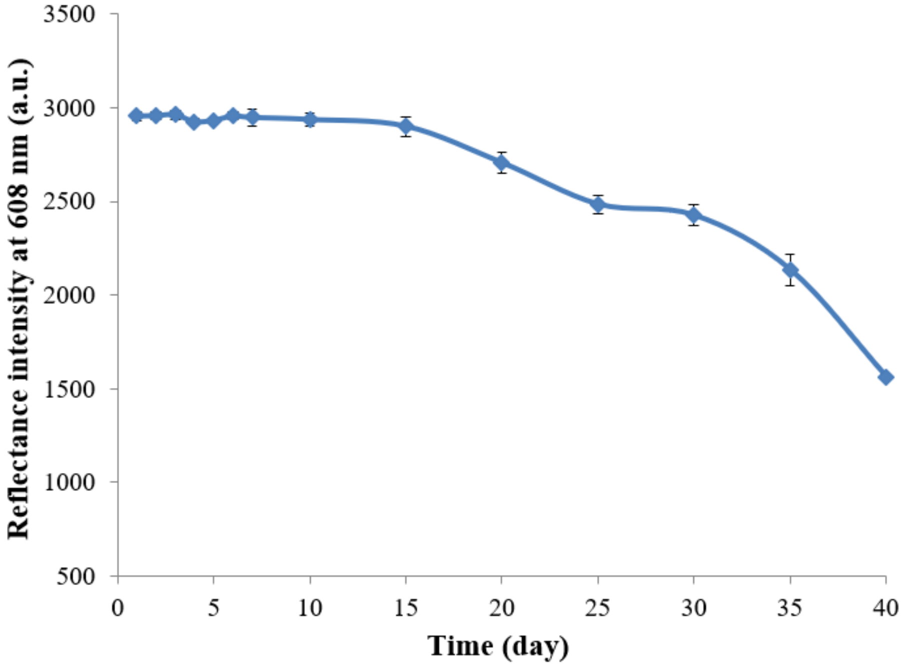

Figure S4. Long-term stability performance of the optical nitrite biosensor based on ruthenium-modified microbial R5-fusion nano-silica over 40 days of experimental period using 20 mg L^{-1} nitrite in 0.1 M K-phosphate buffer (pH 7.0).
